# Levels of brain‐derived neurotrophic factor in patients with multiple sclerosis

**DOI:** 10.1002/acn3.51215

**Published:** 2020-10-08

**Authors:** Yvonne Naegelin, Katharina Saeuberli, Sabine Schaedelin, Hayley Dingsdale, Stefano Magon, Sergio Baranzini, Michael Amann, Katrin Parmar, Charidimos Tsagkas, Pasquale Calabrese, Iris Katharina Penner, Ludwig Kappos, Yves‐Alain Barde

**Affiliations:** ^1^ Neurologic Clinic and Policlinic Departments of Medicine Clinical Research, Biomedicine and Biomedical Engineering University Hospital and University of Basel Basel 4031 Switzerland; ^2^ School of Biosciences Cardiff University Cardiff CF10 3AX United Kingdom; ^3^ Clinical Trial Unit Department of Clinical Research University Hospital Basel Basel 4031 Switzerland; ^4^ Pharma Research and Early Development Roche Innovation Center Basel F. Hoffmann‐La Roche Ltd Basel 4058 Switzerland; ^5^ Department of Neurology University of California, San Francisco San Francisco CA 94158 USA; ^6^ Medical Image Analysis Center (MIAC) AG Basel 4051 Switzerland; ^7^ Department of Biomedical Engineering University of Basel Allschwil 4123 Switzerland; ^8^ Department of Psychology Division of Molecular and Cognitive Neuroscience University of Basel Basel 4055 Switzerland; ^9^ Department of Neurology Medical Faculty Heinrich‐Heine‐University Düsseldorf Düsseldorf 40225 Germany

## Abstract

**Objective:**

To determine the levels of brain‐derived neurotrophic factor (BDNF) in the serum of patients suffering from multiple sclerosis (MS) to evaluate the potential of serum BDNF as a biomarker for MS.

**Methods:**

Using a recently validated enzyme‐linked immunoassay (ELISA) we measured BDNF in patients with MS (pwMS), diagnosed according to the 2001 McDonald criteria and aged between 18 and 70 years, participating in a long‐term cohort study with annual clinical visits, including blood sampling, neuropsychological testing, and brain magnetic resonance imaging (MRI). The results were compared with an age‐ and sex‐matched cohort of healthy controls (HC). Correlations between BDNF levels and a range of clinical and magnetic resonance imaging variables were assessed using an adjusted linear model.

**Results:**

In total, 259 pwMS and 259 HC were included, with a mean age of 44.42 ± 11.06 and 44.31 ± 11.26 years respectively. Eleven had a clinically isolated syndrome (CIS), 178 relapsing remitting MS (RRMS), 56 secondary progressive MS (SPMS), and 14 primary progressive MS (PPMS). Compared with controls, mean BDNF levels were lower by 8 % (p˂0.001) in pwMS. The level of BDNF in patients with SPMS was lower than in RRMS (p = 0.004).

**Interpretation:**

We conclude that while the use of comparatively large cohorts enables the detection of a significant difference in BDNF levels between pwMS and HC, the difference is small and unlikely to usefully inform decision‐making processes at an individual patient level.

## Introduction

Despite considerable efforts, personalized treatment of MS patients is still empirical, largely based on clinical practice and experience. In particular, paucity of readily accessible biomarkers makes it difficult to predict the individual course of the disease and potential treatment response. Because of its multiple “trophic” roles in the nervous system^1^, ^2^, ^3^, ^4^ brain‐derived neurotrophic factor (BDNF) has received considerable attention as a potential biomarker.^3^ In a large number of studies, the levels of BDNF in human serum have been measured in multiple conditions including neurodegenerative and psychiatric diseases.^3^, ^4^ Previous reports have also explored possible links between levels of BDNF in serum and different MS courses.^5^, ^6^ In MS lesions, at the cellular level, beyond neurons, BDNF is primarily present in immune cells including T cells and microglial cells, possibly also in reactive astrocytes.^7^ A “trophic” interaction between infiltrating immune cells and neurons has been repeatedly discussed^7^, ^8^, ^9^, ^10^. This included speculations that the reduced availability of BDNF from immune cells and the resulting loss of neuroprotective effects may contribute to neuro‐axonal degeneration and loss of neurons in chronic forms of MS.^11^ Furthermore, the unexpected observation that fingolimod, a drug frequently administered to MS patients and targeting sphingosine‐1 phosphate receptors, increases the levels of BDNF in rodent neurons has fueled speculations that some of the beneficial effects of the drug may result from increased BDNF levels.^12^

So far, a range of technical issues have complicated BDNF measurements in human serum, with for example six commercially available ELISAs giving different results with the same samples.^13^ In addition, in a considerable majority of previous reports, the cohorts compared have been too small to allow meaningful comparisons. Recently, using a validated ELISA technique, we could show that BDNF measurements in healthy individuals yield sufficiently stable values for meaningful comparisons if cohorts of sufficient sizes can be recruited.^14^

In this study, we compare BDNF levels in a large cohort of MS patients (pwMS) with age‐ and sex‐matched healthy controls (HC) and relate the levels of serum BDNF cross‐sectionally and longitudinally to comprehensive clinical, neuropsychological, and imaging measures to further explore the potential of serum BDNF as biomarker for pwMS.

## Subjects

Data of pwMS and HC were obtained from participants of an ongoing international MS cohort study (only local patients were included) originally designed for a genome‐wide association analysis.^15^ This cohort study was approved in 2003 by the local ethics committee (EKNZ, Ethikkommission Nordwest‐ und Zentralschweiz, Basel, Switzerland). All subjects gave written informed consent.

## Material and Methods

Patients underwent annual comprehensive examinations at baseline (BL) and at annual follow‐up visits (Follow‐up 1, FU1); Follow‐up 2, FU2; and so on for every following visit (cohort study is still ongoing). At each visit, the following assessments were performed: Neurostatus‐UHB,^16^ cognitive tests, questionnaires for fatigue and depression, blood sampling, and standardized Magnetic Resonance Imaging (MRI) scans. In HCs blood samples were only obtained at BL and FU1 (no other assessments were performed). The numbers of pwMS by visit were as follows: BL: 259, FU1: 241, FU2: 208, FU3:181, FU4: 169, FU5: 152, FU6: 144, FU7: 124, FU8: 99, FU9: 94, FU10: 28. The numbers of HC by visit were: BL: 259, FU1: 226.

### Cognition, Fatigue, and Depression

For cognition, Paced Auditory Serial Addition Test (PASAT)^17^ and Symbol‐Digit Modalities Test (SDMT)^18^ were performed at baseline (BL) and annually thereafter. In addition after the third year of follow‐up the Multiple Sclerosis Inventarium Cognition (MUSIC),^19^ Fatigue Scale for Motor functions and Cognition, FSMC^20^ and the depression scale ADS‐L^21^ were performed.

### Magnetic resonance imaging

Head MRI scans were performed on all patients at baseline and then yearly in a 1.5 T scanner (Magnetom Avanto, Siemens Healthineers) equipped with a 12‐element head matrix coil. The yearly scanning protocol included a transversal two‐dimensional (2D) double‐echo proton density‐/T2‐weighted scan with a spatial resolution of 0.98x0.98x3 mm^3^, a sagittal three‐dimensional (3D) T1‐weighted scan (isotropic 1mm^3^ resolution), and a transversal 2D T1‐weighted contrast enhanced spin‐echo scan (0.98x0.98x3 mm^3^).

At each time point white matter lesions (WML) were semi‐automatically segmented on PDw/T2w using intensity thresholding with Amira 3.1.1 (Mercury Computer System) by trained observers according to the standard operating procedures established for the analysis of clinical phase II and phase III trials. The WML volume (WMLV) was then computed for each MRI session.^22^ Normalized volumes of whole brain (NBV), white matter (NWMV), and grey matter (NGMV) were estimated by SIENAX;^23^ deep gray matter structures (thalamus, globus pallidus, and striatum) and hippocampus were segmented using FIRST.^24^ Annualized percentage brain volume change (PBVC) was estimated between BL and FU2 and between BL and FU5/6 with SIENA,^23^ part of FSL.^25^ SIENA; SIENAX and FIRST were used as implemented in the FMRIB software library (FSL version 6.0). The 3D T1w images covered also the upper cervical spinal cord, approximately down to the C3‐5 level. Cervical spinal cord volume was analyzed by cord image analyser (cordial).^26^, ^27^ Segmentation was carried out over a 35‐mm long spinal cord segment starting 27 mm below the cisterna pontis, which corresponds to the spinal cord volume between the foramen magnum and the C2/C3 intervertebral disc.

### Blood sampling

Blood sampling was performed as described before,^14^ and genotyping (including polymorphism rs6265) was done using the Sentrix® HumanHaP550 Bead Chip.^15^ Blood sampling for MS patients was performed annually (up to 10 years after BL), blood sampling for HC only at BL and at FU1. BDNF levels were determined by ELISA using the protocol described previously.^14^ Platelet (thrombocyte) counts (Tc) and the volume percentage of red blood cells in blood (hematocrit, Hct calculated from the number and size of red blood cells) was analyzed with scatter and morphology cytograms as the levels of BDNF depend on both values.^14^

### Statistical analysis

The analyses were performed in R version 3.6.3. All statistical tests are performed two‐tailed.

BDNF levels were log‐normally distributed and therefore log‐transformed prior to all analysis (in the figures the raw data values are presented). Age, sex, Tc, and Hct were included as covariates (if not described differently). For the models, the back‐transformed estimates (mult.effects) are presented together with 95 % confidence intervals (CI). The CIs are estimated on the log‐scale using normal approximation and then back‐transformed by exponentiating.

The association between BDNF and clinical measures (Expanded Disability Status Scale (EDSS), disease course, disease groups and medication), neuropsychological measures (PASAT, SDMT, MUSIC, FSMC, and ADS‐L) as well as MRI measures (brain (whole and selected regions) and spinal cord volume, T2‐lesion volume, atrophy) were all considered, details see below.

The BDNF rs6265 polymorphism was compared (Met/Met vs. all others) using a Wilcoxon rank sum test with continuity correction.

#### BDNF and clinical measures

Log‐transformed BDNF levels at BL were compared between HC and MS patients using a t‐test. BDNF levels at BL were modeled in a linear model. Course of disease was included as factor using HC as reference level. Age, sex, Tc count, and Hct were included as covariates. Therefore estimates refer to the estimated difference in the log‐transformed BDNF‐levels between the respective level of disease course and HC.

For the analysis within the MS patient cohort, disease group and disease duration were included as additional covariates. For disease course RRMS was used as reference level.

The association between BDNF levels at BL and EDSS at BL was modeled as described above. In addition, the association of BDNF (all measurements) and EDSS (all measurements) was modeled using generalized estimating equations (GEE) to account for the within‐subject correlations. Age and sex are included as covariates.

The association between BDNF levels at BL and medication at BL was modeled as described above. For medication the contrast “no medication” versus “medication” was used. In addition RRMS as well as SPMS patients without medication were compared to HC using a t‐test and a linear model including age, sex, Tc and Hct as covariates. There was no sufficient data to account for past treatment or treatment duration.

#### BDNF and neuropsychological measures

The association between BDNF levels and PASAT/SDMT results at BL was assessed in a linear model. The model was adjusted for progressive disease course (PPMS and SPMS) versus relapsing forms (RRMS and CIS), age, sex, Tc count, Hct and education (4 years or less, 5–10 years, 11–15 years, more than 15 years of school education). The association was also assessed at FU1 considering the change in BDNF levels between FU1 and BL.

For the results of MUSIC, ADS‐L and the FSMC components the same models were fitted at FU2, since this was the first available measurement. The same was repeated for the outcome variable at FU2 and the change in the BDNF level between FU2 and FU1.

#### BDNF and MRI measures

The association between BDNF and T2w lesion volumes was assessed using a linear model, where T2‐lesion volume was log‐transformed and treated as a dependent variable. The same was repeated for associations at FU1 and with the change in BDNF levels between FU1 and BL.

The association between BDNF at FU1 and the number of new/enlarging T2w lesions was modeled in a poisson model using GEE to correct for the correlation of measurements in the same individual. The association between BDNF at BL and brain volume (NBV, NGMV, NWMV), the volume of selected brain regions and the volume of the spinal cord was assessed using a linear model. The model was corrected for the covariates disease course (using relapsing MS as a reference level vs. progressive MS), age, sex, Tc count, and Hct. The same was done for the outcome variable at FU1 and the change in the BDNF level between FU1 and BL.

In addition the association between brain volume at FU1 and BDNF change between FU1 and BL was assessed adjusting for the covariates brain volume at BL and age.

Annualized percentage brain volume change (PBVC) was calculated based on measurements of brain volumes at FU2 and at FU5/6 compared to BL. The association between annualized 2‐year brain atrophy and BDNF levels at BL, as well as changes between BL and FU2 were assessed in a linear model, with brain atrophy being used as a dependent variable. The model was also adjusted for the covariate disease course and also applied to the annualized FU5/FU6‐brain atrophy. Some patients had two measurements (FU5 brain atrophy and FU6 brain atrophy) and in these cases, the average of both measurements was used. GEE are used to account for the correlation between the measurements in the same individual.

## Results

In total, 259 patients with MS (pwMS) according to 2001 McDonald criteria^28^ and 259 age and sex‐matched healthy controls (HC) were included into this analysis. Participants were aged 18‐70. The cohort study included all clinical subtypes of MS^28^. There was no significant difference regarding sex, age and the genetic polymorphism rs6265 between pwMS and HC at BL (Table 1).

**Table 1:** Baseline characteristics of MS‐patients and HC

	Healthy controls	MS patients	*P*	MS patient subgroups (disease course)			
				CIS	RR MS	SP MS	PP MS
N (%)	259	259		11 (4.2)	178 (68.7)	56 (21.6)	14 (5.4)
Sex male (%)	81 (31.3)	78 (30.1)	0.849	4 (36.4)	40 (22.5)	27 (48.2)	7 (50)
Age (mean ± (sd))	44.31 (11.26)	44.42 (11.06)	0.909	36.04 (7.95)	41.74 (10.32)	53.77 (8.86)	47.70 (7.22)
Genetic Poly‐ morphism N (%)			0.446				
AA (Met/Met)	10 (4.8)	7 (2.7)		1 (9.1)	5 (2.8)	1 (1.8)	0 (0)
AG (Met/Val)	69 (33.2)	81 (31.8)		1 (9.1)	55 (31.2)	22 (40.0)	3 (23.1)
GG (Val/Val)	129 (62.0)	167 (65.5)		9 (81.8)	116 (65.9)	32 (58.2)	10 (76.9)
Disease Duration inyears(mean ± (sd))	n.a.	8.58 (7.34)		1.70 (1.70)	7.89 (6.48)	12.64 (8.79)	5.93 (6.39)
EDSS (mean ± (sd))	n.a.			1.41 (0.80)	2.61 (1.37)	4.95 (1.35)	4.64 (1.43)
No MS medication N (%)	n.a.	97 (37.5)		8 (72.7)	56 (31.5)	19 (33.9)	14 (100)
MS medication N (%)	n.a.	162 (62.5)		3 (27.3)	122 (68.5)	37 (66.1)	0 (0.0)
Fumaric acid		1 (0.4)		0 (0.0)	1 (0.5)	0 (0.0)	0 (0.0)
Glatiramer acetate		33 (12.7)		0 (0.0)	29 (16.3)	4 (7.2)	0 (0.0)
Interferon β‐1a i.m.		25 (9.7)		3 (27.3)	21 (11.8)	1 (1.8)	0 (0.0)
Interferon β‐1a s.c.		40 (15.4)		0 (0.0)	35 (19.7)	5 (8.9)	0 (0.0)
Interferon β‐1b s.c.		56 (21.6)		0 (0.0)	34 (19.1)	22 (39.3)	0 (0.0)
Mitoxantrone		7 (2.7)		0 (0.0)	2 (1.1)	5 (8.9)	0 (0.0)

CIS, clinically isolated syndrome; RRMS, relapsing remitting MS; SPMS, secondary progressive MS; PPMS, primary progressive MS. *P* values refer to HC versus all MS patients.

**Figure 1:**
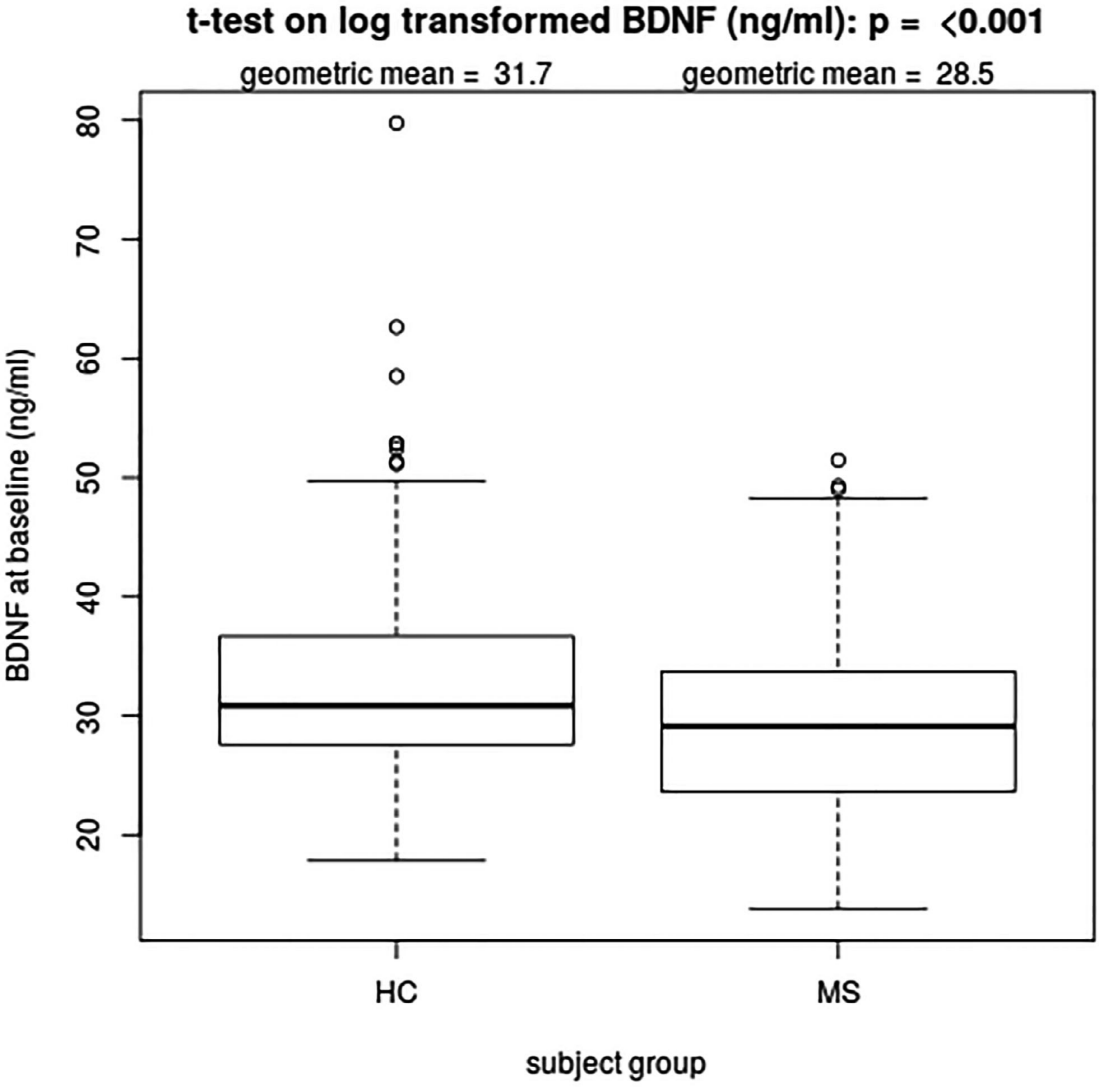
BDNF levels at BL (ng/ml) in MS patients compared to HC (HC taken fromRef. 14). The geometric mean as presented in this figure corresponds to the exponential of the arithmetic mean of log‐transformed BDNF levels. *P*‐value refers to comparison of log‐transformed BDNF values

The median BDNF level at BL was 29.13 ng/ml (mean and sd: 29.41 ± 7.24) in MS patients and 30.86 ng/ml (mean and sd 32.69 ± 8.33) in HC. The BL levels were lower in patients with a secondary progressive course of disease: median 28.49 ng/ml (mean and sd: 27.87 ± 7.55). The summary statistics of BDNF levels at BL are shown in Table 2. BDNF at BL was lower by the factor 0.92 (8 %) in patients with MS compared to HC (p˂0.001, 95 % CI [0.89;0.96]; Fig. 1). Patients with RRMS and SPMS had lower BDNF levels compared to HC (RRMS: *P* = 0.003, 95 % CI [0.90;0.98]; SPMS *P *˂ 0.001, 95 % CI [0.80; 0.91]). SPMS patients had lower levels than RRMS patients (*P* = 0.004, 95 % CI [0.82; 0.96]).

**Table 2:** Summary statistics of BDNF mean values in serum

Group		N	mean	SD	median	IQR
MS Patients	All	259	29.41	7.24	29.13	10.09
	CIS	11	30.13	9.93	30.16	17.57
	RRMS	178	29.52	6.73	29.20	9.45
	SPMS	56	27.87	7.55	28.49	9.86
	PPMS	14	33.67	8.72	32.34	10.08
	All with medication	162	28.43	6.83	28.30	9.70
	All without medication	97	31.05	7.62	29.88	10.51
Healthy Controls		259	32.69	8.33	30.86	9.09

BDNF values indicated in ng/ml. N, Number of patients; SD, standard deviation; IQR, Interquartile range; CIS, Clinically isolated Syndrome; RRMS, Relapsing Remitting MS; SPMS, Secondary progressive MS; PPMS, Primary progressive MS.

**Figure 2:**
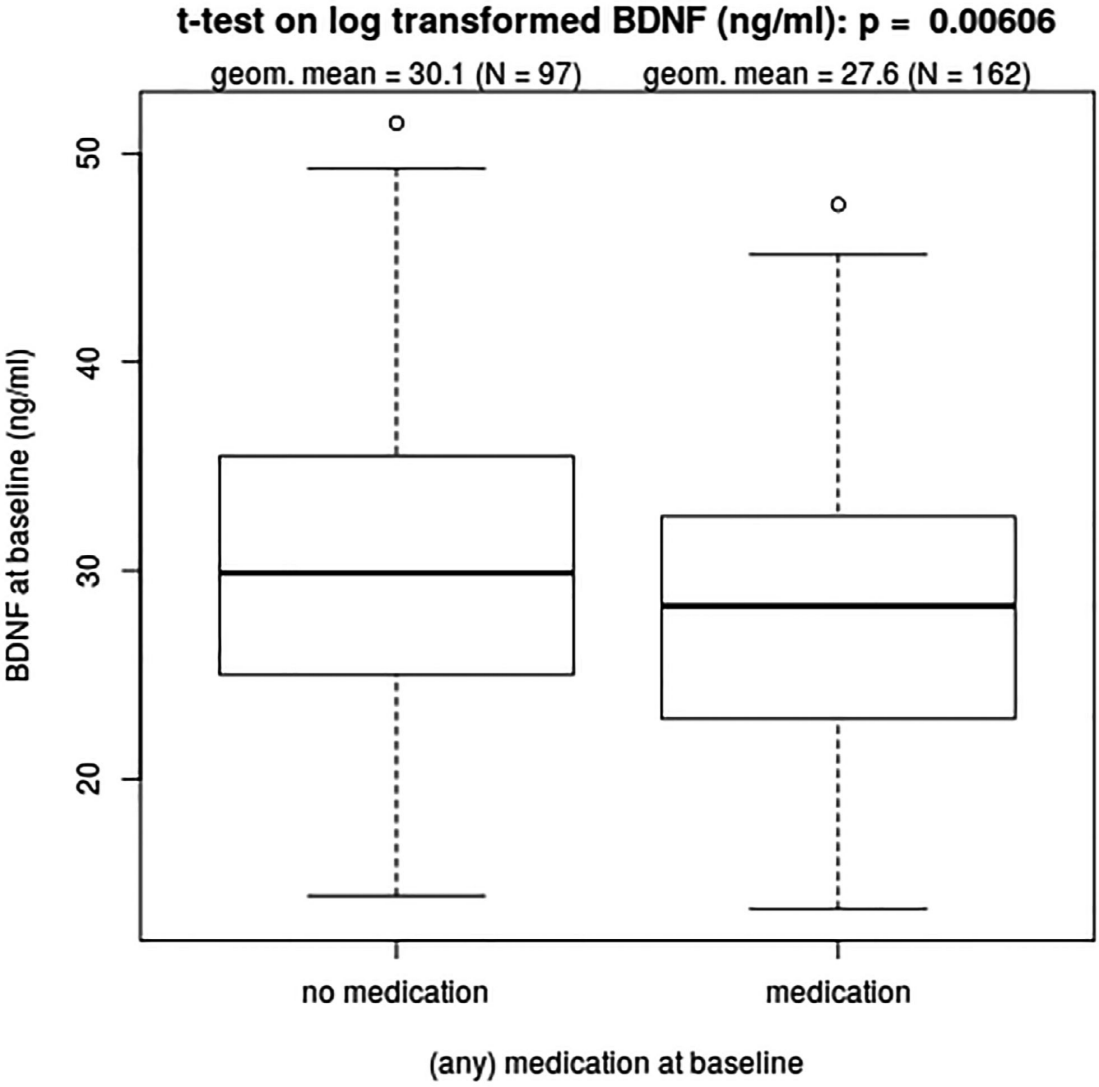
Comparison of log transformed BDNF levels by treatment (*N* = 259)

BDNF at BL was lower in patients with medication (*N* = 162) when compared to untreated patients (*N* = 97) when compared in a simple, t‐test (Fig. 2). However, after adjusting for age, sex, course of disease, disease duration, Hct and Tc count, this was no longer the case (*P* = 0.06, 95 % CI [0.89; 1.00]). There was no difference between untreated RRMS patients and HC at BL (*P* = 0.243, 95 % CI [0.90; 1.03]). However, BDNF levels of untreated SPMS patients were lower than the levels in HC (*P* = 0.026, 95 % CI [0.80; 0.99]).

No significant association between EDSS and BDNF was detected.

With regard to PASAT, no significant association could be detected with BDNF levels. With regard to the association between changes over time, BDNF levels and PASAT (BDNF FU 1 minus BDNF at BL) and PASAT at FU1 for each increase of BDNF by 1 ng/mL within this year, PASAT decreased by −0.33 points (−0.33, *P* = 0.045, 95 % CI [−0.65; −0.01]).

No association between BDNF (change) and the following parameters could be found: SDMT, MUSIC, ADS‐L, FSMC, T2w‐lesion volume, new/enlarging T2w lesions, NBV, NGMV, NWMV, regional brain volumes (Thalamus, Striatum, Globus pallidus, Hippocampus), spinal cord volume, and annualized PBVC.

Concerning the genetic polymorphisms (Met/Met, Val/Met, Val/Val) no significant difference in BDNF levels could be detected when pooling all participants (*P* = 0.269, Table 3). For details of all results see Table S1.

**Table 3:** BDNF levels in ng/ml by genetic polymorphism (PM) and group (MS vs. HC)

Group	PM	N	Q1	Mean	SD	Median	Q3
All	Met/Met	17	22.41	28.61	7.146	27.41	33.26
	Val/Met	150	26.12	31.12	7.643	30.10	34.73
	Val/Val	296	24.68	30.61	8.067	29.88	34.69
	Missing	55	28.27	34.02	7.982	32.39	37.64
HC	Met/Met	10	22.70	28.63	7.575	29.09	33.12
	Val/Met	69	26.80	32.66	8.108	30.74	36.13
	Val/Val	129	27.30	32.46	8.634	30.81	36.60
	Missing	51	28.74	34.14	7.866	32.52	37.64
MS	Met/Met	7	23.98	28.58	7.079	26.15	31.88
	Val/Met	81	23.84	29.81	7.010	29.54	33.82
	Val/Val	167	23.37	29.18	7.311	29.13	33.34
	Missing	4	27.20	32.48	10.592	27.92	33.20

N, number of patients; Q1, 1st quartile; SD, standard deviation; Q3, 3rd quartile.

## Discussion

The main goal of this study was to explore whether or not BDNF levels in MS patients reflect their condition (based on clinical and magnetic resonance imaging variables) with measurements based on a recently developed and validated BDNF ELISA in a large cohort of patients with MS and matched healthy controls.

At the group level we found a significant reduction of BDNF levels in pwMS compared to HC. This reduction was more pronounced in patients with SPMS. These findings at the group level are in line with some previous reports,^5^, ^6^, ^29^ while other studies in smaller cohorts^30^ did not confirm a difference.

No association between BDNF or BDNF change over time could be found in any of the following investigated variables: EDSS, PASAT, SDMT, MUSIC, ADS‐L, FSMC, T2w‐lesion volume, new/enlarging T2w lesions, NBV, NGMV, NWMV, regional brain volumes (Thalamus, Striatum, Globus pallidus, Hippocampus), spinal cord volume, and annualized PBVC.

Concerning the longitudinal changes of BDNF from BL to FU1, 106 (40.9%) of the individuals retested after 12 months had BDNF serum values within 10 % of their original reading (connected by blue lines seen in Fig. S1), 78 (30.1%) individuals showing 10‐20% of changes (green line) and 57 (22%) >20 % changes. This is in line with the previously reported results from HC^14^. Looking at patients with confirmed progression (EDSS 0: +1.5, EDSS 1‐5: +1, EDSS ≥ 5.5: +0.5) over time no clear influence on BDNF levels could be shown (Fig. S2). We conclude that serum BDNF levels are stable over time and do not reflect occurrence of clinical confirmed progression (the latter statement based on a few cases (*N* = 54) only).

In the past, lower serum BDNF levels in patients suffering from a number of neurological conditions compared to HC have been reported (such as depression,^31^ schizophrenia,^32^, ^33^, ^34^, ^35^, ^36^, ^37^, ^38^ Alzheimer’s,^39^, ^40^ or Huntington’s disease.^41^, ^42^ In autism spectrum disorders higher serum levels were found^43^). These studies have been, at least in part, driven by the assumption that BDNF levels in serum may somehow reflect BDNF levels in the brain as diffusion of BDNF from the brain was speculated to explain its presence in the blood and platelets, in analogy with the accumulation of serotonin by platelets. However, there is no direct evidence for a brain‐to‐blood diffusion of BDNF nor has the presence of a BDNF transporter been demonstrated in platelets. Recently, a more plausible explanation for the accumulation of BDNF in human platelets has been presented with the demonstration that megakaryocytes express the BDNF gene at significant levels and fill up platelets with the protein,^44^ as is the case for several other growth factors accumulated in platelets.^45^ Conversely, if the brain were the source of BDNF in serum, its concentration would be expected to be higher in the cerebrospinal fluid (CSF), as is the case with neurofilament light chains.^46^ Similar to neurofilaments, BDNF is primarily contained in neurons in the central nervous system^47^ and it is conceivable that its levels might be influenced in this specific compartment as a result of lesions accompanying MS; as has been demonstrated to be the case for neurofilament light chains. It remains still unclear to us if reliable techniques for measuring BDNF in CSF do exist, but as far as known from the existing literature the levels of BDNF in CSF are very low.^48^ The changes in BDNF levels in blood or serum are more likely to reflect platelet activation and degranulation rather the neuronal damage in conditions such as MS, mood disorders, or neurodegeneration.^49^ In this study, we tried to overcome some possible limitations: first of all we used a previously described ELISA protocol,^14^ did also correct for platelet counts and the hematocrit value and compared a sufficient large cohort of patients with MS and sex‐ and age‐matched healthy volunteers. In addition the possibility that the genetic polymorphism rs6265 could explain the difference in serum levels could be excluded. The replacement of valine by methionine in the pro‐domain of BDNF has received considerable attention given its association with decreased performance in tests of episodic and recall memory.^50^, ^51^ As indicated in Table 3, no significant differences could be observed whereby the sample size could be too small for any firm conclusions to be drawn. In addition we only examined rs6265, one of the many SNPs in the vicinity of this gene. Beyond the technical problems linked with the use of commercially available BDNF ELISAs sample size are also likely to explain why in numerous studies exploring correlations between BDNF levels and the rs6265 polymorphism, no clear conclusions could be drawn.^52^, ^53^, ^54^

Medication was considered to be a possible confounder and could potentially have influenced the results as BDNF at BL was lower in patients with medication (Fig. 2) when compared in a simple t‐test but after adjusting for age, sex, course of disease, disease duration, Hct and Tc count this was no longer the case. In addition BDNF levels of untreated SPMS patients were lower than the levels in HC, even when adjusted for age, therefore we assume that medication was not the only responsible factor for the observed difference in BDNF serum levels, but there was no sufficient data to account for past treatment or treatment duration.

Although the numbers of patients included in this study should have been sufficient for reliable conclusions on a group level, any conclusion on a subgroup level (e.g., patients with confirmed progression over time) could not be reliably drawn.

From a clinical perspective the difference of serum BDNF – although significant at the group level – is relatively small and frequent overlap exists between BDNF values in pwMS and HC at the individual level, further limiting the value of BDNF levels as an aid to diagnosis or as a prognostic marker for patients with MS.

In conclusion, the overall outcome of this study is that *serum* levels of BDNF, even when measured in large cohorts using a validated BDNF ELISA, probably neither reflect nor predict disease progression and/or activity, and therefore are unlikely to be useful as a reliable serum biomarker for pwMS to guide decision‐making processes at an individual level.

## Author Contributions

**YN** is responsible for the concept and the design of this study, developed and validated the immunoassay and performed all BDNF measurements. She is the PI of the cohort study from which the data are derived, and planed the statistical analysis with SS. She wrote the first draft and revised the manuscript. **KS** supervised the performance of the immunoassays and revised the manuscript. **SS** performed all statistical analysis and revised the manuscript. **HD** revised the manuscript. **SM** analyzed imaging data and contributed to the statistical analysis of MRI data. He revised the manuscript. **SB** provided the genetic data (analysis) and revised the manuscript. **MA**, **KP,** and **CT** analyzed imaging data and revised the manuscript. **PC** and **IKP** gave input for neuropsychological testing of the patient cohorts and revised the manuscript. **LK** supervised the clinical cohort study and revised the manuscript. **YAB** supervised the development and the performance of the immunoassay and revised the manuscript.

## Conflict of Interest

YN, KS, SS, HD, SM, SB, MA, KP, CT, and YAB: have nothing to disclose. **PC** reports honoraria for speaking at scientific meetings, serving at scientific advisory boards and consulting activities from Abbvie, Actelion, Almirall, Bayer‐Schering, Biogen Idec, Celgene, EISAI, Genzyme, Lundbeck, Merck Serono, Novartis, Pfizer, Teva, Sanofi‐Aventis and grants from Swiss Multiple Sclerosis Society and from Swiss National Research Foundation, outside the submitted work. **IKP** reports honoraria for speaking at scientific meetings, serving at scientific advisory boards and consulting activities from Adamas Pharma, Almirall, Bayer Pharma, Biogen, Celgene, Desitin, Genzyme, Merck, Novartis, Roche, Teva and grants from German MS Society, Teva, Celgene, Novartis, outside the submitted work. **LK**s’ Institution (University Hospital Basel) received steering committee, advisory board and consultancy fees in the last 3 years, which were used exclusively for research support at his department, from Actelion, Alkermes, Almirall, Bayer, Biogen, Celgene/Receptos, df‐mp, Excemed, GeNeuro SA, Genzyme, Japan Tobacco, Merck, Minoryx, Mitsubishi Tanabe Pharma, Novartis, Roche, Sanofi Genzyme, Santhera, Teva, Vianex and license fees for Neurostatus‐UHB products. The Research of the MS Center in Basel has been supported by grants from Bayer, Biogen, the European Union, Novartis, Roche Research Foundations, the Swiss MS Society, Inoswiss and the Swiss National Research Foundation.

## Supporting information


**Figure S1.** BDNF serum levels at BL and FU1. Serum BDNF values of participants at BL (n = 259) and FU1 (n = 241) visit. Measurements of the same subject are connected by a colored line to indicate percentage change in BDNF values between visits. Samples are separated on the x‐axis by age in years. A: RRMS and B: SPMS.

**Figure S2.** BDNF serum levels over time in patients with confirmed progression (all visits marked as a black circle, visits with confirmed progression marked red).

**Table S1.** Overview of all statistical analysis and results (green: result statistically significant/ Magenta: result statistically not significant). 1 = Adjusted for age, sex, Tc, Hct. DC = Disease Course. DD = Disease Duration. E = Education. If not otherwise indicated the values are BL. BL = Baseline, FU1 = Follow‐up 1, FU2 = Follow‐up 2, FU3 = Follow‐up 3, FU4 = Follow‐up 4, FU5 = Follow‐up 5, FU6 = Follow‐up 6. PBVC = Percentage Brain Volume Change, NBV = Normalized (Total) Brain Volume, NGMV = Normalized Grey Matter Volume, NWMV = Normalized White Matter Volume.
