# Supplementary figures and images for: Levels of brain‐derived neurotrophic factor in patients with multiple sclerosis
Source: Ann Clin Transl Neurol. 2020 Oct 8;7(11):2251–61. doi: 10.1002/acn3.51215 (PMC7664260; doi:10.1002/acn3.51215)

- only one measurement, N = 18 (6.9 %)
- 0-10% change, N = 106 (40.9 %)
- 10-20% change, N = 78 (30.1 %)
- 20-30% change, N = 34 (13.1 %)
- more than 30% change, N = 23 (8.9 %)

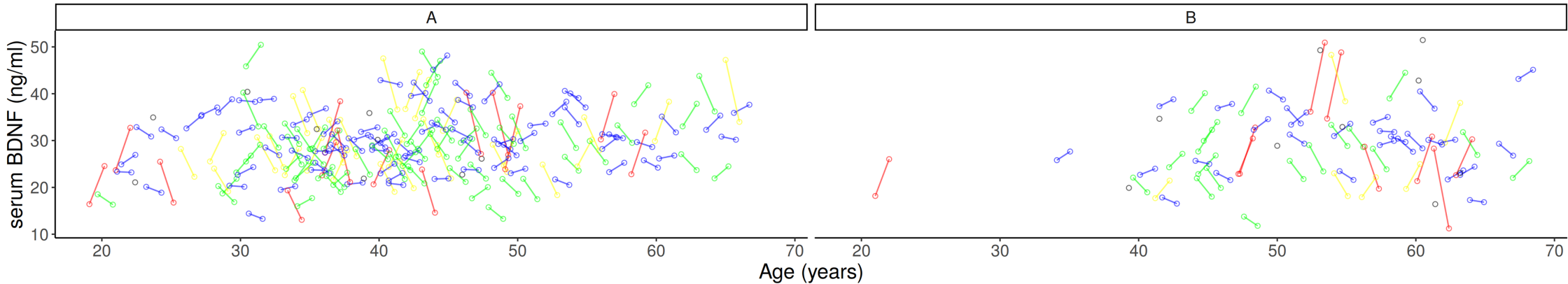

Supplement: Supplementary file 1 — Figure S1. BDNF serum levels at BL and FU1. Serum BDNF values of participants at BL (n = 259) and FU1 (n = 241) visit. Measurements of the same subject are connected by a colored line to indicate percentage change in BDNF values between visits. Samples are separated on the x‐axis by age in years. A: RRMS and B: SPMS. [file ACN3-7-2251-s001.pdf]

confirmed progression ●

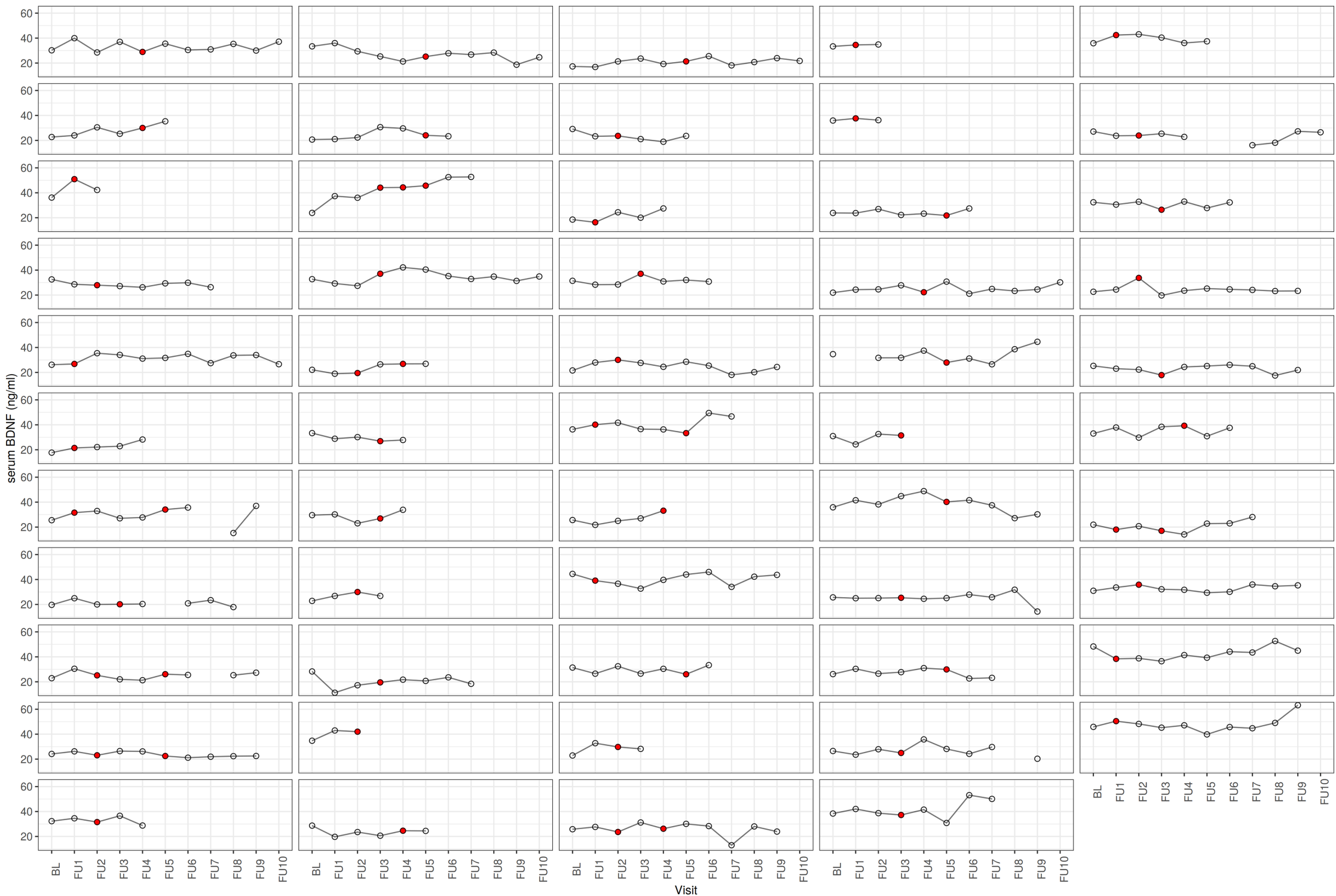

Supplement: Supplementary file 2 — Figure S2. BDNF serum levels over time in patients with confirmed progression (all visits marked as a black circle, visits with confirmed progression marked red). [file ACN3-7-2251-s002.pdf]
