# Supplementary material for: Levels of brain‐derived neurotrophic factor in patients with multiple sclerosis
Source: Ann Clin Transl Neurol. 2020 Oct 8;7(11):2251–61. doi: 10.1002/acn3.51215 (PMC7664260; doi:10.1002/acn3.51215)
Supplement: Supplementary file 3 — Table S1. Overview of all statistical analysis and results (green: result statistically significant/ Magenta: result statistically not significant). 1 = Adjusted for age, sex, Tc, Hct. DC = Disease Course. DD = Disease Duration. E = Education. If not otherwise indicated the values are BL. BL = Baseline, FU1 = Follow‐up 1, FU2 = Follow‐up 2, FU3 = Follow‐up 3, FU4 = Follow‐up 4, FU5 = Follow‐up 5, FU6 = Follow‐up 6. PBVC = Percentage Brain Volume Change, NBV = Normalized (Total) Brain Volume, NGMV = Normalized Grey Matter Volume, NWMV = Normalized White Matter Volume. [file ACN3-7-2251-s003.docx]

Supplemental Table 1. Overview of all statistical analysis and results

| **Hypothesis** | **Re-sult yes/**  **no** | **Esti-mate** | **Mult**  **Ef-fect** | **[95% CI]** | **p** | **Additional Information** |
| --- | --- | --- | --- | --- | --- | --- |
| BDNF is lower in patients with MS compared to HC | yes | -0.08 | 0.92 | [0.89;0.96] | ˂0.001 | Adjusted for: 1 (see legend) |
| BDNF is lower in patients with RRMS compared to HC | yes | -0.07 | 0.94 | [0.90;0.98] | 0.003 | Adjusted for: 1  Influence of medication could not be excluded |
| BDNF is lower in patients with SPMS compared to HC | yes | -0.16 | 0.85 | [0.80;0.91] | ˂0.001 | Adjusted for: 1 |
| BDNF is lower in patients with SPMS compared to RRMS | yes | -0.12 | 0.89 | [0.82;0.96] | 0.004 | Adjusted for: 1 and DD |
| BDNF is lower in patients with medication (N=162) than in untreated patients (N=97) | no | -0.06 | 0.94 | [0.89;1.00] | 0.06 | Adjusted for: 1 and DD |
| BDNF is lower in untreated RRMS patients compared to HC | no | -0.04 | 0.96 | [0.90;1.03] | 0.243 | Adjusted for: 1 |
| BDNF is lower in untreated SPMS patients compared to HC | yes | -0.12 | 0.89 | [0.80;0.99] | 0.026 | Adjusted for: 1 |
| BDNF is lower in patients with strongly progressive disease courses compared to all other patients | no | -0.09 | 0.92 | [0.79;1.06] | 0.235 | Adjusted for: 1 and DD |
| Association of BDNF and EDSS | no | 0.01  -0.01 | 1.01  0.99 | [0.99;1.03]  [0.93;1.06] | 0.246  0.784 | Adjusted for: 1, DC, DD  N=252  **GEE** (N=1696), not adjusted for Tc and Hct |
| Association of BDNF and PASAT | no | 0.03 |  | [-0.18;0.25] | 0.761 | Adjusted for: 1 and E.  Older patients (-0.16, p=0.031, 95 % CI [-0.30;-0.02]), patients with a progressive form of MS (-3.85, p=0.034, 95 % CI [-7.39;-0.31]) as well as patients with less education (-7.69 if only 5-10 years of education, p˂0.001, 95 % CI [-11.78;-3.60]) performed worse than younger patients, patients with relapsing forms of MS or patients with better education. |
| Association of BDNF change (FU1-BL) and PASAT FU1 | yes | -0.33 |  | [-0.65;-0.01]) | 0.045 | Adjusted for: 1 and E.  For each increase of BDNF of 1 ng/ml within this year PASAT decreased -0.33 points |
| Association of BDNF and SDMT | no | -0.08 |  | [-0.36;0.20] | 0.574 | Adjusted for: 1 and E.  Older patients (-0.47, p˂0.001, 95 % CI [-0.65;-0.28]) and patients with less education (-8.87 if only 5-10 years of education, p=0.002, 95 % CI [-14.52;-3.23]) performed worse than younger patients and patients with better education. |
| Association of BDNF change (FU1-BL) and SDMT FU1 | no | -0.09 |  | [-0.53;0.35] | 0.686 | Adjusted for: 1 and E. |
| Association of BDNF FU2 and MUSIC FU2 | no | -0.06 |  | [-0.17;0.05] | 0.280 | Adjusted for: age, sex, E.  Older patients (-0.11, p=0.008, 95 % CI [-0.19;-0.03]), male patients (-2.51, p=0.003, 95 % CI [-4.13;-0.89]) and patients with less education (-3.62 if only 5-10 years of education, p˂0.004, 95 % CI [-6.05;-1.18]) performed worse than younger patients, female patients and patients with better education. |
| Association of BDNF change (FU2-FU1) and MUSIC FU2 | no | -0.13 |  | [-0.29;0.03] | 0.114 | Adjusted for: age, sex, E. |
| Association of BDNF FU2 and ADS-L FU2 | no | 0.996 |  | [0.99;1.01] | 0.431 | Adjusted for: age, sex, E.  Male patients had higher scores than female patients (0.867, p=0.037, 95 % CI [0.759;0.991]). |
| Association of BDNF change (FU2-FU1) and ADS-L FU2 | no | 0.997 |  | [0.984;1.01] | 0.640 | Adjusted for: age, sex, E. |
| Association of BDNF FU2 and FSMC (total score) FU2 | no | -0.11 |  | [-0.52;0.30] | 0.586 | Adjusted for: age, sex, E.  Older patients (0.52, p˂0.001, 95 % CI [0.22;0.83]) as well as patients with less education (6.61 if 11-15 years of education, p=0.048, 95 % CI [0.10;13.13] and 13.31 if only 5-10 years of education, p=0.005, 95 % CI [4.18;22.45]) had higher scores than younger patients and patients with better education. |
| Association of BDNF change (FU2-FU1) and FSMC (total score) FU2 | no | -0.10 |  | [-0.7;0.50] | 0.739 | Adjusted for: age, sex, E. |
| Association of BDNF FU2 and FSMC (motor score) FU2 | no | -0.04 |  | [-0.26;0.17] | 0.690 | Adjusted for: age, sex, E.  Older patients (0.25, p=0.002, 95 % CI [0.09;0.41]), patients with a progressive form of MS (6.23, p=0.001, 95 % CI [2.57;9.89]) as well as patients with less education (6.09 if 5-10 years of education, p=0.013, 95 % CI [1.32;10.86]) had higher scores than younger patients, patients with relapsing forms of MS and patients with better education. |
| Association of BDNF change (FU2-FU1) and FSMC (motor score) FU2 | no | -0.05 |  | [-0.36;0.26] | 0.763 | Adjusted for: age, sex, E. |
| Association of BDNF FU2 and FSMC (cognitive score) FU2 | no | -0.07 |  | [-0.29;0.15] | 0.531 | Adjusted for: age, sex, E. Older patients (0.27, p=0.001, 95 % CI [0.11;0.43]), as well as patients with less education (7.22 if 5-10 years of education, p=0.004, 95 % CI [2.33;12.12]) had higher scores than younger patients and patients with better education. |
| Association of BDNF change (FU2-FU1) and FSMC (cognitive score) FU2 | no | -0.05 |  | [-0.37;0.27] | 0.743 | Adjusted for: age, sex, E. |
| Association of BDNF and T2w lesion volume | no |  | 0.99 | [0.97;1.02] | 0.526 | Adjusted for: DC. |
| Association of BDNF change (FU1-BL) and T2w lesion volume FU1 | no |  | 0.99 | [0.95;1.03] | 0.593 | Adjusted for: DC. |
| Association of BDNF and new/enlarging T2w lesions | no |  | 1.00 | [0.97;1.03] | 0.87 | **GEE**  Adjusted for: DC |
| Association of BDNF FU1 and new/enlarging T2w lesions | no |  | 0.99 | [0.97;1.02] | 0.67 | **GEE**  Adjusted for: DC |
| Association of BDNF and  - NBV  - NGMV  - NWMV | no  no  no | 0.18  -0.04  0.21 |  | [-1.46;1.82]  [-0.95;0.87]  [-0.69;1.12] | 0.833  0.935  0.642 | Adjusted for: 1 and DC.  Older patients had less brain volumes than younger ones (total: -4.22, p˂0.001, 95 % CI [-5.33;-3.12] / gray matter: -2.91, p˂0.001, 95 % CI [-3.52;-2.30] / white matter: -1.31, p˂0.001, 95 % CI [-1.92;-0.70]). |
| Association of BDNF change (FU1-BL) and  - NBV FU1  - NGMV FU1  - NWMV FU1 | no  no  no | 0.42  -0.21  0.41 |  | [-1.89;2.72]  [-1.51;1.10]  [-0.86;1.68] | 0.723  0.758  0.527 | Adjusted for: 1 and DC. |
| Association of BDNF and regional brain volumes:  - Thalamus  - Striatum  - Globus pallidus  - Hippocampus | no  no  no  no | 0.00  -0.01  -0.00  0.00 |  | [-0.03;0.04]  [-0.05;0.03]  [-0.01;0.01]  [-0.01;0.02] | 0.823  0.724  0.667  0.412 | Adjusted for: 1 and DC.  Older patients had smaller volumes than younger patients (Thalamus: -0.06, p˂0.001, 95 % CI [-0.09;-0.04] /Striatum: -0.12, p˂0.001, 95 % CI [-0.14;-0.09] /Globus pallidus: -0.01, p˂0.001, 95 % CI [-0.01;-0.00] /Hippocampus: no significant difference). In male patients the volumes were smaller than in female patients in Thalamus -0.91, p=0.004, 95 % CI [-1.52;-0.30], Striatum -0.85, p=0.013, 95 % CI [-1.52;-0.18] and Hippocampus -0.27, p=0.004, 95 % CI [-0.45;-0.09]). |
| Association of BDNF change (FU1-BL) and regional brain volumes FU1:  - Thalamus  - Striatum  - Globus pallidus  - Hippocampus | no  no  no  no | 0.01  0.05  0.01  -0.00 |  | [-0.04;0.07]  [-0.01;0.10]  [-0.00;0.01]  [-0.02;0.01] | 0.639  0.110  0.244  0.924 | Adjusted for: 1 and DC. |
| Association of BDNF and  spinal cord volume | no | 0.00 |  | [-0.00;0.01] | 0.128 | Adjusted for: 1 and DC.  Progressive patients (spinal cord volumes: -0.18, p˂0.001, 95 % CI [-0.28;-0.08]) had lower spinal cord volumes than patients with relapsing forms of MS. Male patients had increased volumes compared to female patients (spinal cord volumes: 0.26, p˂0.001, 95 % CI [0.17;0.35]). |
| Association of BDNF change (FU1-BL) and  spinal cord volume FU1 | no | -0.00 |  | [-0.01;0.01] | 0.758 | Adjusted for: 1 and DC. |
| Association of BDNF (BL) and annualized PBVC within two years (BL and FU2) | no | -0.00 |  | [-0.01;0.01] | 0.693 | Adjusted for: DC. |
| Association of BDNF (FU2) and annualized PBVC within two years (BL and FU2) | no | -0.01 |  | [-0.02;0.00] | 0.181 | Adjusted for: DC. |
| Association of BDNF change (FU2-BL) and annualized PBVC within two years (BL and FU2) | no | -0.01 |  | [-0.03;0.01] | 0.205 | Adjusted for: DC. |
| Association of BDNF (BL) and annualized PBVC within 5-6 years (BL and FU5/FU6) | no | -0.00 |  | [-0.01;0.00] | 0.25 | **GEE**  Adjusted for: DC. |
| Association of BDNF (FU5/FU6) and annualized PBVC within 5-6 years (BL and FU5/FU6) | no | -0.00 |  | [-0.01;0.01] | 0.97 | **GEE**  Adjusted for: DC. |

Supplemental Table 1: Analysis&Results Summary (green: result statistically significant / Magenta: result statistically not significant)

1=Adjusted for age, sex, Tc, Hct. DC=Disease Course. DD=Disease Duration. E=Education. If not otherwise indicated the values are BL. BL=Baseline, FU1=Follow-up 1, FU2=Follow-up 2, FU3=Follow-up 3, FU4=Follow-up 4, FU5=Follow-up 5, FU6=Follow-up 6. PBVC=Percentage Brain Volume Change, NBV=Normalized (Total) Brain Volume, NGMV=Normalized Grey Matter Volume, NWMV=Normalized White Matter Volume.
